# Drug discovery by a basic research scientist

**DOI:** 10.3389/fmolb.2022.1062346

**Published:** 2022-11-03

**Authors:** William A. Eaton

**Affiliations:** Laboratory of Chemical Physics, National Institute of Diabetes and Digestive and Kidney Diseases, National Institutes of Health, Bethesda, MD, United States

**Keywords:** sickle cell, HbS, polymerization, drug discovery, drug screening

## Abstract

I was fortunate to do my military service during the Vietnam era as a medical officer at the National Institutes of Health (NIH) in Bethesda, Maryland. My first research at NIH was concerned with making a variety of optical measurements on nucleic acid bases and proteins, including single crystal spectra in linearly polarized light and near infrared circular dichroism, interpreting the spectra using molecular orbital and crystal field theories. What I do now is drug discovery, a field at the opposite end of the scientific spectrum. This article gives a brief account of my transition from spectroscopy to sickle cell hemoglobin polymerization to protein folding to drug discovery for treating sickle cell disease. My lab recently developed a high throughput assay to screen the 12,657 compounds of the California Institute of Biomedical Research ReFrame drug repurposing library. This is a precious library because the compounds have either been FDA approved or have been tested in clinical trials. Since the 1970s numerous agents have been reported in the literature to inhibit HbS polymerization and/or sickling with only one successful drug, hydroxyurea, and another of dubious value, voxelotor, even though it has been approved by the FDA. Our screen has discovered 106 anti-sickling agents in the ReFrame compound library. We estimate that as many as 21 of these compounds could become oral drugs for treating sickle cell disease because they inhibit at concentrations typical of the free concentrations of oral drugs in human serum.

Thanks to the legendary 20th century chemist, Linus Pauling, sickle cell disease was the first disease to be recognized as being caused by an abnormal molecule. Pauling’s 1949 paper on sickle cell hemoglobin (HbS) gave birth to “molecular medicine,” i.e., understanding disease pathogenesis at the molecular level (Pauling et al., 1949; Eaton, 2003). After almost 20 years of basic research focused primarily on the physics and physical chemistry of sickle cell hemoglobin (HbS) polymerization (Eaton, 2020; Eaton, 2022), I changed the focus my research area in the early 1990s to protein folding (Eaton, 2021). By introducing nanosecond pulsed lasers to initiate protein folding, I and my colleagues improved the time resolution in kinetic experiments by over five orders of magnitude (Jones et al., 1993). These new methods allowed us to resolve the kinetics of secondary structure formation and polypeptide collapse for the first time and show that a small protein could fold in less than 1 microsecond (Eaton et al., 2000; Kubelka et al., 2006). The importance of the work is that our experimental results provided a critical test of the validity of all-atom molecular dynamics simulations of protein folding, which, if accurate, contain everything one would ever want to know about the mechanism of folding for a particular protein. We also improved the time resolution of single molecule fluorescence experiments, which permitted the first measurements of transition path times. The transition path is the rare and very brief event in a molecular trajectory when the molecule crosses the free energy barrier separating thermodynamic states, and in the case of protein folding contains all the mechanistic information on how a protein folds and unfolds (Chung et al., 2012; Chung and Eaton, 2018).

My interest in sickle cell disease began with the use of a microspectrophotometer based on a Leitz polarizing microscope to measure the orientation of the hemes in the fibers that form inside red cells from the polymerization of HbS, an instrument very similar to the microspectrophotometer I assembled in Robin Hochstrasser’s lab as a graduate student (Eaton and Hochstrasser, 1967; Eaton and Hochstrasser, 1968). In 1972, James Hofrichter arrived in the lab as a post-doctoral fellow to work with Elliot Charney after finishing his PhD thesis research with John Schellman at the University of Oregon. Hofrichter became interested in what I was doing, which was the beginning of a close collaboration until his retirement in 2008 and continued on a part-time basis until his untimely death in 2021. Hofrichter was an expert in optics and made several important improvements to my microspectrophotometer. Our measurements showed that the plane of the porphyrins is approximately perpendicular to the fiber axis, providing support for Stuart Edelstein’s model of the fiber based on the helically twisted double strands found by Warner Love in the X-ray structure of deoxyHbS (Hofrichter et al., 1973; Wishner et al., 1975; Dykes et al., 1978).

Sickle cell research had just started to become a popular subject in hematology because of the increased research funding provided by the 1972 National Sickle Cell Disease Control Act, which also attracted many biochemical and biophysical research scientists to the field. One of the great advantages of working at NIH is that I did not have to write a grant proposal; I could immediately change my research direction from molecular spectroscopy to sickle cell disease, which I did after receiving an enthusiastic response to my presentation of our sickle red cell polarized absorption results at Max Perutz’s Royal Society Hemoglobin meeting in London in February 1973. When I returned, I purified HbS from patient blood that I obtained at Howard University Hospital in Washington, DC and with Hofrichter prepared very concentrated solutions (>200 mg/cc) of deoxyHbS. At ice temperatures, the solution remained liquid; at room temperature the solution formed a viscous gel that was birefringent when observed between the crossed polarizers of my Leitz microscope. This optical property permitted us to study the kinetics of HbS polymerization with a temperature jump, albeit a very slow jump, from ice temperature to room temperature in about 1 min. These experiments permitted our discovery of a lag phase prior to the appearance of birefringence, which we called a delay period. Birefringence requires that the fibers be at least partially aligned, so it was not clear that the appearance of birefringence corresponded to polymerization of HbS or to alignment of fibers that followed HbS polymerization. The question was answered when Philip Ross, from the neighboring Laboratory of Molecular Biology, joined us to measure the kinetics with his calorimeter. Ross observed the start of heat absorption at exactly the same time as the appearance of birefringence. With these two methods we discovered the extraordinary kinetics of HbS polymerization, characterized by a delay period with a 90 kcal/mol activation energy and the highest concentration dependence ever measured for a molecular process—30th power (Hofrichter et al., 1974).

Because of my medical education, I was able to immediately realize the clinical significance of our findings (Hofrichter et al., 1974; Eaton et al., 1976). These included the 1) recognition that the delay could permit the vast majority of cells to escape the smallest vessels of the tissues before fibers form to cause vaso-occlusion, which makes the disease survivable 2) the proposal that a small dilution of hemoglobin S with its very large concomitant increase in the delay time would be therapeutic, and 3) the severity of sickle cell disease and its various milder sickle syndromes, such as HbSC disease and sickle beta + thalassemia, could be explained with the simple postulate that the delay time relative to the transit time through the micro-circulation of the tissues is the primary determinant of clinical severity. Factors which decrease the delay time, such as fever, or increase the transit time, such as infection that increases the number of adherent white cells, increase severity, while the opposite decreases clinical severity. These concepts have all been supported by subsequent research. A demanding set of experiments by a visiting scientist to the lab from the University of Parma, Andrea Mozzarelli, suggested that at the oxygen pressures in the tissues the vast majority of cells have delay times longer than transit times, so that sickling *in vivo* is far from equilibrium (Mozzarelli et al., 1987). Mozzarelli’s experiments were carried out on a minutes time scale, while transit times through the smallest vessels of most tissues occur in seconds. A recent extensive theoretical investigation of sickling probability at the much shorter *in vivo* times strongly support Mozzarelli’s findings (Figure 1). (Henry et al., 2020) The data in Figure 1 show that were polymerization at equilibrium most cells would be sickled in all tissues even for sickle trait, the benign heterozygous condition.

**FIGURE 1:**
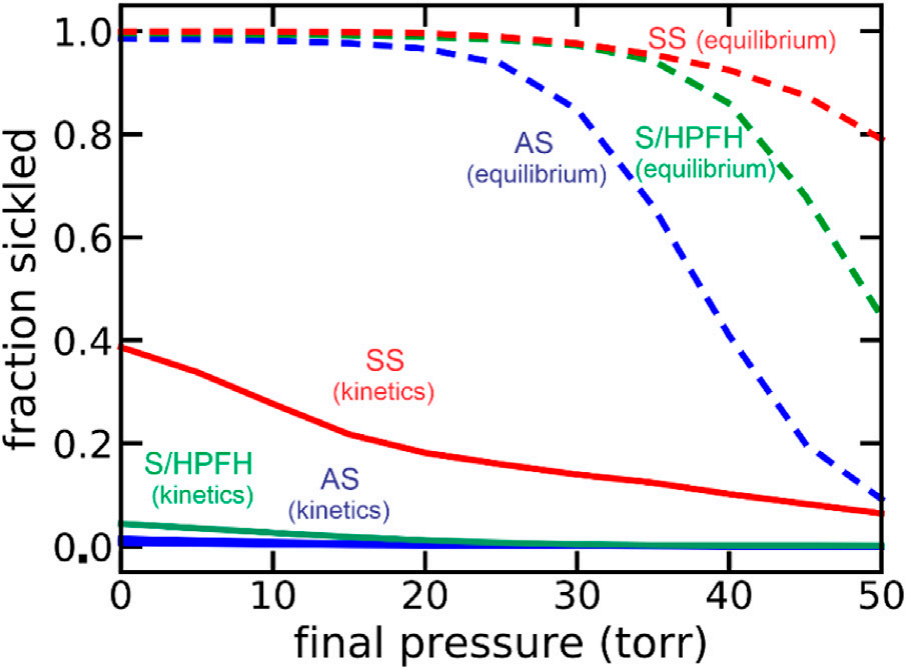
Theoretical calculation of fraction sickled at equilibrium at each oxygen pressure and with the pressure decreasing at an *in vivo* rate of 50 torr/sec (kinetics): sickle cell disease (SS, 95% HbS, red), the asymptomatic double heterozygous of condition of sickle cell disease with pancellular persistence of fetal hemoglobin (S/HPFH, 70% HbS, 30% HbF, green), and the heterozygous condition, sickle trait (AS, 40% HbS, 60% HbA, blue).

The demonstration that a small dilution of hemoglobin S would be therapeutic has been demonstrated by the success of hydroxyurea in reducing the frequency of sickle cell crises. Hydroxyurea, approved by the FDA in 1998, acts by inducing fetal hemoglobin synthesis to dilute HbS and increase the delay time, allowing more cells to escape the narrow vessels of the tissues before sickling occurs (Figure 2) (Sunshine et al., 1978; Eaton and Hofrichter, 1995; Eaton and Bunn, 2017). In addition to the kinetic studies, research with Hofrichter and Ross on the thermodynamics, together with the theoretical work of Allen Minton, led to a description of an HbS gel as a two-phase system similar to a crystal-solution equilibrium (Ross et al., 1977). These studies also explained the solubility of mixtures of HbS with normal and fetal hemoglobins (Sunshine et al., 1979; Bunn et al., 1982) and the oxygen dependence of HbS solubility with an extension of the two-state allosteric model of Monod, Wyman, and Changeux (MWC) to include a tertiary conformational pre-equilibrium (Sunshine et al., 1982; Henry et al., 2002; Viappiani et al., 2004; Viappiani et al., 2014; Henry et al., 2015; Henry et al., 2020).

**FIGURE 2:**
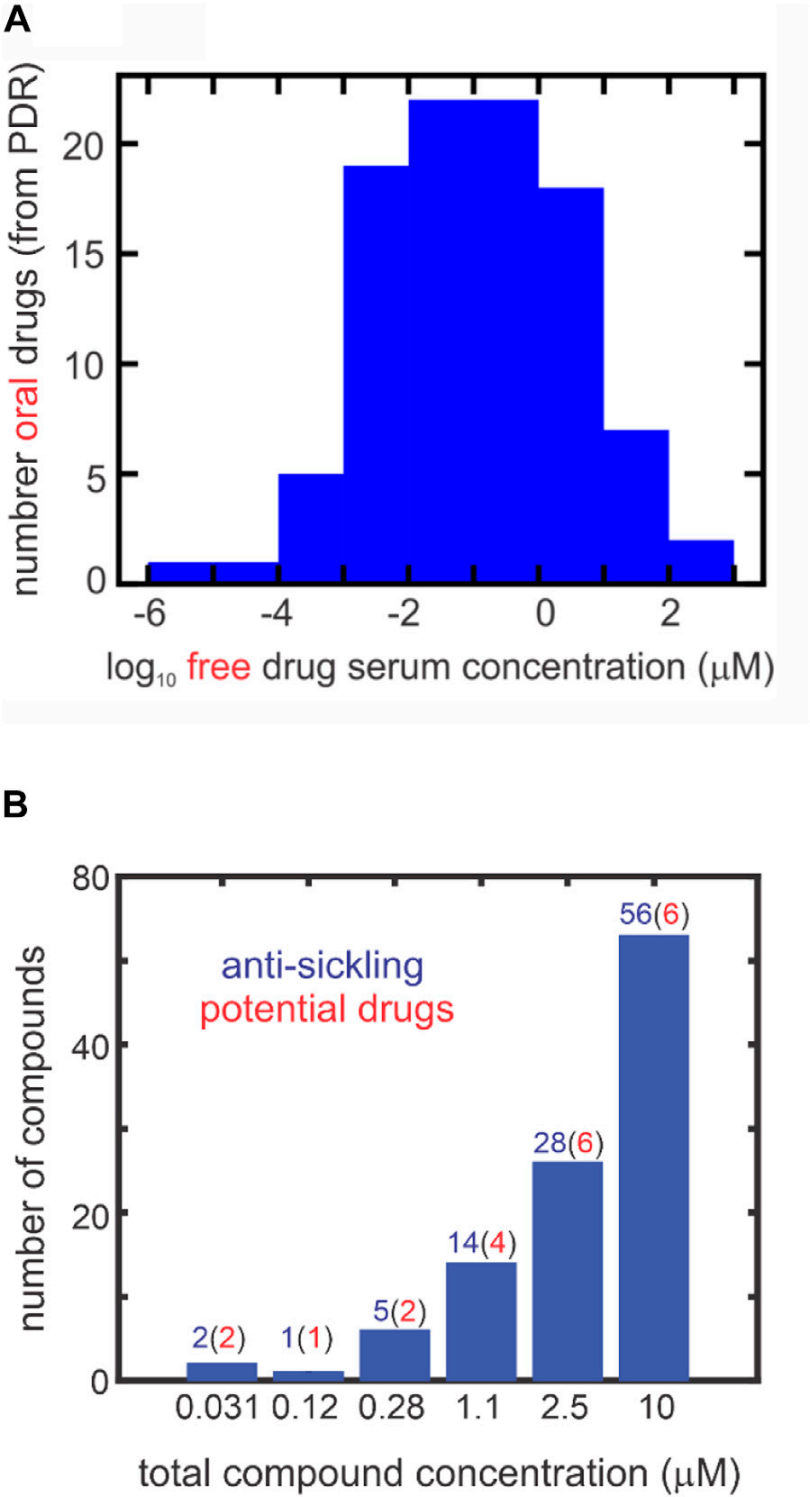
Comparing inhibitory concentrations in assay and oral drug concentrations in the PDR (one column figure). **(A)** Distribution of 97 oral *free* drug concentrations (C_max_) in the 2015 version of the PDR. The free concentration was given either explicitly in the PDR or obtained from the given total serum C_max_ and the percentage bound to serum proteins. **(B)** Distribution of ReFrame total compound concentrations with statistically significant inhibition at each concentration defined by the difference between the fraction sickled for the test compound compared to the cell suspension with no compound (the negative control). The red numbers in parentheses result from multiplying the number (in blue) of compounds by the fraction of oral drugs with that free concentration or higher from the distribution in panel **(A)**. For inhibitory mechanisms other than those resulting from binding to hemoglobin, the total compound concentrationis also the free concentration. For compounds that inhibit by binding to hemoglobin, the free concentration is less than the total concentration. In the 2000-fold diluted blood used for the assay, the hemoglobin molecule is at a concentration of ∼1 μM, while the molar concentration of red cells is ∼4 fM.

My interest in returning to sickle cell research was aroused in 2004, when I attended a sickle cell meeting at NIH organized by Francis Collins, then NIH Director. The FDA approved hydroxyurea for treating sickle cell disease in 1998, but it was only partially successful and remained the only FDA approved drug; the only new drug proposed at the meeting was Aes-103, which was abandoned after it failed clinical trials, possibly for the same reason that voxelotor fails to decrease the frequency of sickle cell crises (Henry et al., 2021; Inusa et al., 2021). My NIDDK colleague, Dan Camerini, also attended the meeting and urged me to return to sickle cell research to use what I had learned from 20 years of basic research on the subject to find additional drugs. My reaction to his suggestion was to think about using a kinetic assay, which is the most relevant to the pathophysiology, to carry out large scale screening for discovering new drugs, rather than attacking a specific target. With very few exceptions (Nakagawa et al., 2022), all of the very large number of drug discovery projects for sickle cell disease based on inhibiting polymerization of HbS, the root cause of the pathology in sickle cell disease, were focused on a specific target (Mehanna, 2001; Olubiyi et al., 2019). The most frequent targeting approach has been to bind a drug to the non-polymerizing R quaternary conformation of HbS (Sunshine et al., 1982; Eaton and Hofrichter, 1990; Henry et al., 2020) to reduce the population of the polymerizing T quaternary conformation (Nakagawa et al., 2014; Safo and Kato, 2014; Pagare et al., 2022). The only FDA-approved drug that resulted from this approach is voxelotor. It was approved because it increased hemoglobin levels in sickle cell disease patients. However, it has had little or no effect on the frequency of the extremely painful vaso-occlusive crises, which is of greatest concern to patients (Howard et al., 2021; Inusa et al., 2021). We have recently shown that preferential binding to the R conformation does indeed markedly reduce sickling, but HbS molecules with the drug bound deliver little or no oxygen, explaining its failure to reduce crisis frequency. An important but subtle issue pointed out in Henry et al. (2021) is that this approach has a much better chance of working if the drug binds and dissociates very rapidly, which does not happen with voxelotor.

Nothing happened until 2006 when Jeffrey Smith started as a post-doctoral fellow with me. Smith was a highly unusual and extremely talented young man, having earned 4 academic degrees at the University of Pennsylvania—a bachelor’s degree in bioengineering, a bachelor’s degree in economics, a master’s degree in bioengineering, and a master of business administration from the Wharton School of Business—all in 4 years and one summer and a grade point average of 4.99/5.0. I came to know Smith because I sponsored the last 2 years of his thesis research with Christopher Dobson at the University of Cambridge in a joint NIH/Cambridge PhD program. In just 9 months he did what an excellent post-doctoral fellow would accomplish in 2–3 years. He developed and applied an assay based on laser photodissociation of carbon monoxide in sickle trait cells to create deoxyhemoglobin in less than one second and an automated data collection and analysis program to determine the sickling delay time for individual red cells. He used the assay in a 96-well plate format to screen a library of 2,500 compounds. With the parting remark that “it is now technician’s work,” he abruptly resigned his post-doctoral fellowship to take a job with the consulting firm, McKinzie.

The project progressed very slowly for the next 10 years, in part because I was focused on protein folding and in part because the Smith laser photolysis assay was very low throughput. But we did show that a small increase in cell volume by ionophores had the large predicted effect on the delay time of cell sickling (Li et al., 2017). In 2017, I became aware of a powerful and user-friendly commercial instrument, a “Lionheart” automated microscope system of Biotek, now owned by Agilent Technologies. With this instrument deoxygenation of sickle trait cells was carried out with nitrogen using a gas-controller that measured the partial pressure of oxygen in a humidified chamber containing a 384 well plate. This instrument had the additional advantage that we did not need to add the very strong reducing agent (sodium dithionite) that could potentially alter test compounds, which was needed in the laser photolysis assay to scavenge oxygen (Dunkelberger et al., 2018; Henry et al., 2021).

Thanks to my NIH colleague, John Tisdale, who told the Gates Foundation administrators about our assay during a visit to NIH by Bill Gates, we gained access to the Gates-funded library of the California Institute of Biomedical Research (Calibr) at the Scripps Institute. The library contained 12,657 compounds and is a precious collection because the compounds have either been approved by the FDA as drugs or have been tested in clinical trials. Consequently, any compound found to be anti-sickling in our assay at concentrations that can be achieved in the serum is a potential drug and could be rapidly tested in clinical trials without extensive pre-clinical studies that are expensive and time consuming, so long as the known side effects are not particularly deleterious to sickle cell disease patients. Since there are five distinct anti-sickling mechanisms (Eaton and Bunn, 2017), with four of the five being detected in our new assay, we were optimistic that we would discover many new anti-sickling compounds. Using our new assay we in fact discovered 106 of the 12,657 Calibr compounds to be ant-sickling (Metaferia et al., 2022). The complete list of anti-sickling compounds can be found in the recently published paper by Metaferia et al. (2022) Figure 2B is an interesting summary of the results of Metaferia et al. (2022) by showing the number of compounds at each concentration that are anti-sickling. These are free concentrations, as no protein was added to the cell suspension. Assuming that the distribution of free concentrations in our collection of 106 is the same as the distribution of free concentrations in the serum of oral drugs in the Physician’s Desk Reference (Figure 2A), we estimated that as many as 21 of the 106 compounds could be drugs. The probable number at each concentration is shown in red in parentheses at each concentration in Figure 2B.

There are obvious caveats to the analysis based solely on serum concentrations such as the fact that the drug must be taken for the lifetime of the patient and the drug’s side effects may not be tolerated by sickle cell disease patients. Nevertheless, we regard our results as a major breakthrough in drug therapy for sickle cell disease and will serve as a catalyst for many others to develop one or more of these 106 anti-sickling compounds into effective drugs. Our next step in the project is to determine the mechanism of inhibition for all 106 compounds. This will determine what drugs can be given in combination to have additive effects because they attack different targets. In our assay, blood is diluted 1000-fold, so we are developing an assay using whole blood, which will allow us to directly determine the total (bound plus free drug) serum concentration for each level of inhibition.

I should conclude with a comment about the difference I have experienced between basic biophysical studies and drug discovery. My research in basic biophysical studies has been curiosity driven and focused on developing and applying new kinds of experiments to solve outstanding problems, with theory playing a key role in interpreting experimental results and suggesting new experiments. Drug discovery has been very different. It is goal driven, where empirical findings dominate and the same experiment is performed over and over again, with theory playing a relatively minor role. Drug discovery research is very high risk, but if it results in alleviating patient suffering it is very high payoff.

## Data Availability

Publicly available datasets were analyzed in this study. This data can be found here: Metaferia et al. (2022).
